# Pathogenesis of Tobacco-Associated Lung Adenocarcinoma Is Closely Coupled with Changes in the Gut and Lung Microbiomes

**DOI:** 10.3390/ijms231810930

**Published:** 2022-09-18

**Authors:** Casey T. Finnicum, Zahraa Rahal, Maya Hassane, Warapen Treekitkarnmongkol, Ansam Sinjab, Rhiannon Morris, Yuejiang Liu, Elizabeth L. Tang, Sarah Viet, Jason L. Petersen, Philip L. Lorenzi, Lin Tan, Joseph Petrosino, Kristi L. Hoffman, Junya Fujimoto, Seyed Javad Moghaddam, Humam Kadara

**Affiliations:** 1 Avera Institute for Human Genetics, Sioux Falls, SD 57108, USA; 2 Department of Translational Molecular Pathology, The University of Texas MD Anderson Cancer Center, Houston, TX 77030, USA; 3 Faculty of Medicine, American University of Beirut, Beirut P.O. Box 11-0236, Lebanon; 4 College of Natural Sciences, University of Texas at Austin, Austin, TX 78705, USA; 5 UTHealth Houston Graduate School of Biomedical Sciences, The University of Texas MD Anderson Cancer Center, Houston, TX 77030, USA; 6 Department of Physics, University of Illinois at Urbana-Champaign, Urbana, IL 61801, USA; 7 Metabolomics Core Facility, Department of Bioinformatics and Computational Biology, The University of Texas MD Anderson Cancer Center, Houston, TX 77030, USA; 8 Department of Molecular Virology and Microbiology, Baylor College of Medicine, Houston, TX 77030, USA; 9 Department of Pulmonary Medicine, The University of Texas MD Anderson Cancer Center, Houston, TX 77030, USA

**Keywords:** microbiome, lung adenocarcinoma, smoking, 16S rRNA sequencing

## Abstract

Microbial dysbiosis has emerged as a modulator of oncogenesis and response to therapy, particularly in lung cancer. Here, we investigate the evolution of the gut and lung microbiomes following exposure to a tobacco carcinogen. We performed 16S rRNA-Seq of fecal and lung samples collected prior to and at several timepoints following (nicotine-specific nitrosamine ketone/NNK) exposure in *Gprc5a^−/−^* mice that were previously shown to exhibit accelerated lung adenocarcinoma (LUAD) development following NNK exposure. We found significant progressive changes in human-relevant gut and lung microbiome members (e.g., *Odoribacter*, *Alistipes*, *Akkermansia*, and *Ruminococus*) that are closely associated with the phenotypic development of LUAD and immunotherapeutic response in human lung cancer patients. These changes were associated with decreased short-chain fatty acids (propionic acid and butyric acid) following exposure to NNK. We next sought to study the impact of *Lcn2* expression, a bacterial growth inhibitor, given our previous findings on its protective role in LUAD development. Indeed, we found that the loss of *Lcn2* was associated with widespread gut and lung microbiome changes at all timepoints, distinct from those observed in our *Gprc5a^−/−^* mouse model, including a decrease in abundance and diversity. Our overall findings apprise novel cues implicating microbial phenotypes in the development of tobacco-associated LUAD.

## 1. Introduction

Groundbreaking advances in investigating alterations in distinct microbial niches within the human body ecosystem have unraveled the imperative impact of microbiota dysbiosis (disruption to the microbiota homeostasis) as an emerging key modulator of oncogenesis and response to therapy [1]. A plethora of evidence has crowned the gut microbiota as a major regulator of immunity and host inflammation [2]. It is now conceived that the gut’s microbial ecosystem can affect systemic immune homeostasis and inflammation, increasing susceptibility to malignancies outside the GI tract [1,3]. Furthermore, it was shown that the gut microbiome modulates the response to immune checkpoint inhibitors in lung cancer patients, rendering probiotics and fecal microbiota transfers viable means to improve the efficacy and reduce the toxicity of immunotherapies [4,5]. Indeed, gut dysbiosis caused by antibiotics prior to immunotherapy can affect response to treatment [6]. While healthy lung tissues were long perceived as sterile entities, it was recently shown that certain microbial species reside in the lungs, impacting the microenvironment and thereby affecting predisposition to lung diseases and oncogenesis [7].

The roles and abundance of various microbial species are apt to fluctuations influenced by several exogenous triggers such as smoking [8]. Lung adenocarcinoma (LUAD), that is associated with smoking, remains a leading cancer diagnosis worldwide, warranting new strategies to identify the earliest events that drive this fatal malignancy [9]. We previously reported that mice with a knock-out of G-protein coupled receptor 5A (*Gprc5a*^−/−^), in contrast to wild-type (WT) littermates, develop preneoplasias and LUADs at three and seven months, respectively, following exposure to nicotine-specific nitrosamine ketone (NNK), a tobacco carcinogen, offering a unique opportunity to map the temporal evolution of this malignancy [10]. We further found progressive elevation in LCN2, an antimicrobial protein, during inflammation and LUAD development. We also reported that *Gprc5a*^−/−^; *Lcn2*^−/−^ mice exhibited markedly increased lung oncogenesis concomitant with elevated proinflammatory phenotypes when compared to littermates with intact *Lcn2* [11], suggesting that the upregulated expression of LCN2 during early phases of lung oncogenesis is likely a host defense mechanism (against genetic insults, tobacco smoke, and/or inflammation) that helps to limit protumor inflammatory cues, and exerts protective and host defense roles against LUAD pathogenesis.

However, it is still unclear how the host microbiome (gut and lung) may be implicated in the development of tobacco-associated LUAD. To address this gap, we sought to investigate the evolution of the gut and lung microbiomes following exposure to NNK, a tobacco carcinogen causally linked to lung tumorigenesis in humans [12]. We probed the temporal evolution of the gut and lung microbiome during LUAD development in *Gprc5a*^−/−^ and *Gprc5a*^−/−^; *Lcn2*^−/−^ animals and identified contextual microbial cues that are plausibly implicated in the pathogenesis of the disease.

## 2. Results

### 2.1. Gut and Lung Microbiome Changes during the Phenotypic Evolution of Tobacco Carcinogen-Associated LUAD in Gprc5a^−/−^ Animals

**Figure 1:**
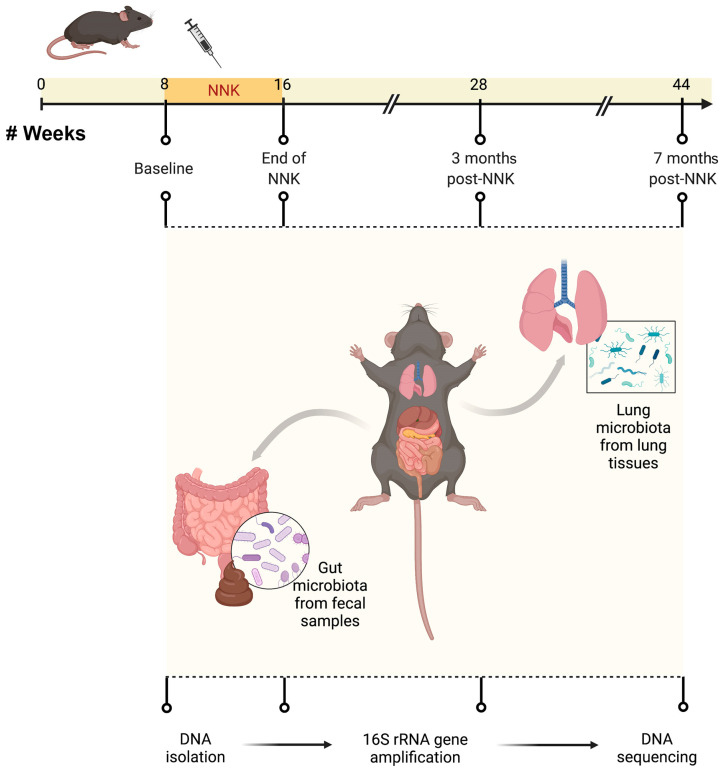
Experimental design and timeline. Eight-week-old *Gprc5a*^−/−^ and *Gprc5a*^−/−^; *Lcn2*^−/−^ mice were divided into four groups (8–10 mice per genotype per time point, matched age, 1:1 male to female ratio) and injected intraperitoneally with 50 mg/kg of body weight NNK three times per week for eight weeks (150 mg/kg total per week) dissolved in PBS. Mice were sacrificed at baseline (prior to NNK treatment) immediately after completion of NNK treatment (end of NNK) and at 3 and 7 months following NNK treatment. Lungs and fecal pellets were collected for DNA extraction, 16S rRNA gene amplification, and subsequent DNA sequencing. NNK: nicotine-specific nitrosamine ketone.

**Figure 2:**
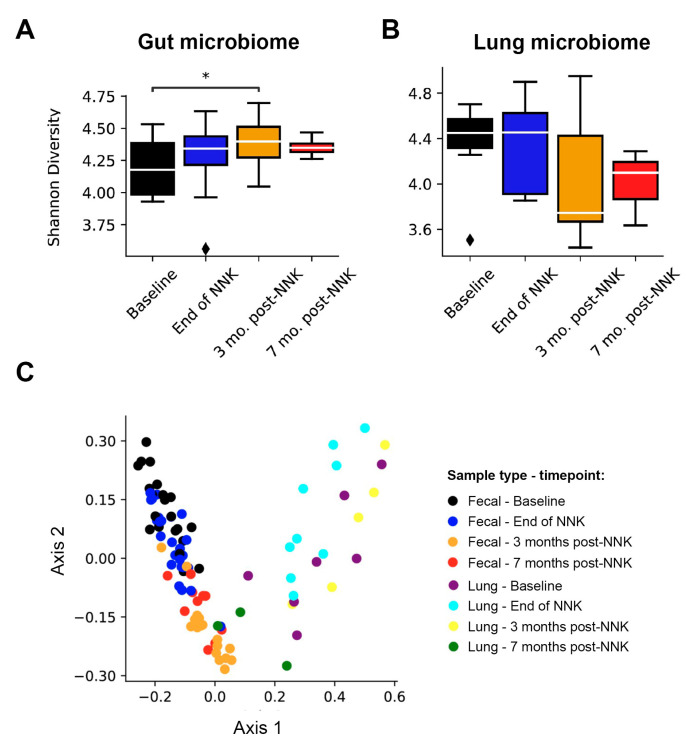
Gut and lung microbiome changes during the phenotypic evolution of tobacco carcinogen-associated LUAD in *Gprc5a*^−/−^ animals. (**A**). Gut microbiome α-diversity evaluated using the Shannon index of *Gprc5a*^−/−^ fecal samples collected at baseline, end of NNK, three-, and seven months post-NNK. (**B**). Lung microbiome α-diversity evaluated using the Shannon index of *Gprc5a*^−/−^ lung tissues collected at baseline, end of NNK, three-, and seven months post-NNK. Shannon indices were calculated using the species-level OTUs. Differences in alpha diversity among the various time points were investigated using an ANOVA and Tukey’s post hoc test where necessary. *, *p* < 0.05; ♦, Outlier. (**C**). Bray–Curtis and OTU-based clustering of fecal and lung samples showing segregation based on sample type (axis 1) and timepoint (baseline, end of NNK, three- and seven months post-NNK; (axis 2). The Bray–Curtis dissimilarity matrix was used as input to the principal coordinate analysis to visualize the data. Clustering observed in the data was tested for statistical significance using an AMOVA with 10,000 iterations. NNK: nicotine-specific nitrosamine ketone.

We previously found that *Gprc5a*^−/−^ mice, when exposed to tobacco carcinogen, develop accelerated premalignant lesions (hyperplasias and adenomas) compared to wild-type littermates and harbor in their tumors somatic activating mutations in *Kras* G12D that are highly pertinent to human LUAD in smokers [10,13,14,15]. To begin to understand the temporal microbial changes associated with tobacco carcinogen exposure in *Gprc5a*^−/−^ mice, we performed microbiome profiling by 16S ribosomal RNA sequencing (16S-seq) of fecal samples at different time points prior to and following NNK exposure (end of NKK, three months, and seven months post-NNK) (Figure 1). We found significant differences in the Shannon indices amongst the different time points of NNK exposure in *Gprc5a^−/−^* gut microbial samples (F-stat = 3.63, *p* = 1.70 × 10^−2^, Figure 2A), suggesting changes in the gut microbiome alpha diversity following tobacco carcinogen exposure. A significant increase in alpha diversity was most conspicuous at three months after exposure to NNK compared to baseline measurements (*p* = 0.0134 of Tukey’s HSD test for pairwise comparison), with a tendency to return to baseline by seven months following exposure to NNK (Figure 2A).

We next interrogated changes in Gprc5a^−/−^ mice lung microbiome in response to exposure to tobacco carcinogen (Figure 1). Variations in lung microbial alpha diversity were observed at different timepoints of NNK exposure, albeit not reaching statistical significance (Figure 2B).

Next, we assessed microbiome composition at different timepoints of NNK exposure from the gut and lung-derived microbial samples via a principal coordinate analysis (PCoA). Indeed, this ordination technique showed the distinct clustering of gut and lung microbiomes relative to each other and among all tested timepoints of NNK exposure (*p* < 1 × 10^−5^ of the overall AMOVA test; Figure 2C), suggesting a shift in the beta diversity of microbial species in response to tobacco carcinogen exposure. Of particular note is more scattered lung microbial alpha and beta diversities when compared to the gut (Figure 2B,C), possibly related to the fact that these microbial changes are local to the lung, i.e., they are spatially closely associated with lung tumors that exhibit intra-group heterogeneity as well lung inflammation. Additionally, the heterogenous alpha diversity of the lung microbiome may, in part, be influenced by its low biomass [16]. These data suggest that the pathogenesis of LUADs in Gprc5a^−/−^ mice following exposure to tobacco carcinogen is paralleled with broad changes in gut and lung microbiota profiles, including shifts in alpha and beta diversities.

### 2.2. Specific Phyla and Genera Orchestrate Shifts in Gut and Lung Microbial Ecosystems during LUAD Pathogenesis

**Figure 3:**
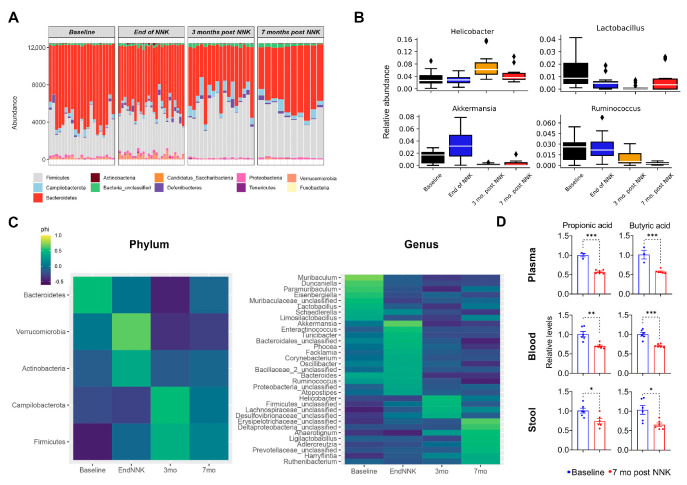
Specific phyla and genera drive shifts in the gut microbial ecosystem during LUAD pathogenesis in *Gprc5a*^−/−^ animals. (**A**) Changes in abundance of bacterial phyla at different timepoints of NNK exposure. (**B**) Changes in gut select bacterial genera at different timepoints of NNK exposure identified through SIMPER analysis and analyzed via a Kruskal–Wallis test with FDR correction. The corrected *p*-value threshold was 0.05; ♦, Outlier. (**C**) Heat map of the indicator analysis conducted to identify phyla (**left**) and genera (**right**) that are good “indicators” of microbial ecosystems at different NNK exposure timepoints. Indicator species were significantly more abundant and present in all samples belonging to one group and absent or with low abundance in the other group. The *p*-values obtained from the permutations of the indicator analysis were subsequently corrected via FDR procedures. (**D**) Plasma, blood, and stool bacterial metabolites were statistically compared between baseline and seven months post-NNK timepoints using the student’s *t*-test. *, *p* < 0.05; **, *p* < 0.01; ***, *p* < 0.001; NNK: nicotine-specific nitrosamine ketone.

Marked gut and lung microbiomes clustering among all four timepoints of NNK exposure prompted us to interrogate individual taxa associated with the observed variation in microbial composition. This was performed using a SIMPER analysis to identify taxa that explain dissimilarities in microbial compositions among the various time points of NNK exposure, followed by testing for significant differential enrichment at both the phylum and genus levels. We found significantly and progressively changing bacterial phyla during the pathogenesis of tobacco carcinogen-associated LUAD, including the abolishment of Verrucomicrobia in the gut (Figure 3A, Supplementary Figure S1A). We also noted a progressive increase in the phylum Firmicutes coupled with a decrease in Bacteroidetes in the gut following exposure to NNK (Supplementary Figure S1A). Our SIMPER analysis revealed 22 genera-level operational taxonomic units (OTUs), which explain at least 1% of the variation between two exposure time points in the gut of *Gprc5a^−/−^* mice (Table 1), 18 of which survived further follow-up significance analyses with Metastats and FDR correction (Figure 3B, Supplementary Figure S1B). For instance, we found significant progressive changes in the abundance of microbial genera, such as elevation in the tumor-promoting bacteria *Helicobacter*, as well as an attenuation of *Lactobacillus*, *Akkermansia*, and *Ruminococcus*, which were previously demonstrated to be associated with a favorable response to cancer therapy (Figure 3B).

Similar analyses performed on the *Gprc5a*^−/−^ lung microbial ecosystem identified 30 unique genera through the initial SIMPER analysis, with five genera surviving multiple testing correction procedures (Table 2, Supplementary Figure S2A,B).

Next, to identify microbial taxa that could discriminate across the four NNK exposure groups (baseline, end of NNK, three- and seven months post-NNK), the indicator species analysis (ISA) was performed (Figure 3C, Supplementary Figure S2C). Indicator species were significantly more abundant and present in all samples belonging to one group and absent or with low abundance in the other group. For instance, among others, *Akkermansia*, which is associated with favorable responses to immunotherapy [4], was found to be an indicator species at the end of NNK, then decreased progressively to be replaced by the tumor-promoting bacteria *Helicobacter* [17], which stood out at three months post-NNK in the gut microbiome (Figure 3C). On the other hand, *Alistipes* and *Escherichia/Shigella* increased with time to dominate the lung microbial ecosystem by seven months following exposure to NNK (Supplementary Figure S2C). Thus, it is plausible that NNK exposure leads to acute changes in the microbiome abundance (e.g., increase in *Akkermansia* by the end of NNK) that could be distinct from changes occurring in association with tumorigenesis (e.g., decrease in *Akkermansia* at three months post-NNK). We then sought to probe the levels of short-chain fatty acids (SCFAs) secreted by microbes, given their known associations with microbial imbalances and immune homeostasis. Indeed, we found that microbial changes were accompanied by significantly decreased circulating and gut levels of SCFAs (propionic and butyric acids) post-tobacco carcinogen exposure and during LUAD development (*p* < 0.05; Figure 3D).

All in all, our findings insinuate that exposure to tobacco carcinogen elicits progressive changes in the gut and lung microbiome taxonomic composition that are closely associated with the phenotypic development of LUAD.

### 2.3. Global Fecal Microbiome Changes Associated with Lcn2 Expression during Lung Oncogenesis

**Figure 4:**
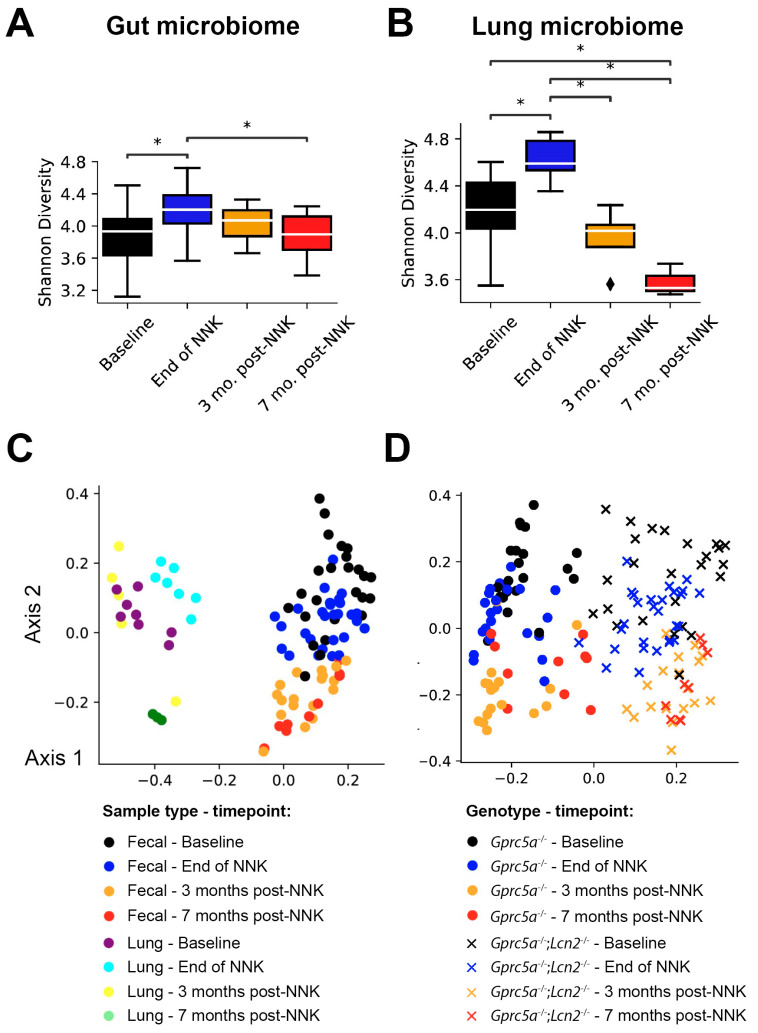
Gut and lung microbiome changes during the phenotypic evolution of tobacco carcinogen-associated LUAD in *Gprc5a*^−/−^; *Lcn2*^−/−^ animals. (**A**) Gut microbiome α-diversity evaluated using the Shannon index of *Gprc5a*^−/−^; *Lcn2*^−/−^ fecal samples collected at baseline, end of NNK, three-, and seven months post-NNK. (**B**) Lung microbiome α-diversity evaluated using the Shannon index of Gprc5a^−/−^; *Lcn2*^−/−^ lung tissues collected at baseline, end of NNK, three-, and seven months post-NNK. Shannon indices were calculated using the species-level OTUs. Differences in alpha diversity among the various time points were investigated using an ANOVA and Tukey’s post hoc test where necessary. *, *p* < 0.05; ♦, Outlier. (**C**) Bray–Curtis and OTU-based clustering of fecal and lung samples showing segregation based on sample type (axis 1) and timepoint (baseline, end of NNK, three- and seven months post-NNK; axis 2). (**D**) Bray–Curtis and OTU-based clustering of fecal samples showing segregation based on genotype (axis 1) and timepoint (baseline, end of NNK, three- and seven months post-NNK; axis 2). The Bray–Curtis dissimilarity matrix was used as input to the principal coordinate analysis to visualize the data. Clustering observed in the data was tested for statistical significance using an AMOVA with 10,000 iterations. NNK: nicotine-specific nitrosamine ketone.

As we previously showed that *Gprc5a^−/−^* mice with knock-out of the antimicrobial immunomodulator lipocalin 2 (*Gprc5a*^−/−^; *Lcn2*^−/−^) are more susceptible to nicotine-specific carcinogen-mediated lung cancer development compared to similarly exposed *Gprc5a*^−/−^ with intact *Lcn2*, we sought to investigate the microbial evolution following NNK exposure in this mouse model [11]. Indeed, significant changes in gut microbiome alpha diversity were observed following tobacco carcinogen exposure (F-stat = 5.55, *p* = 1.69 × 10^−3^; Figure 4A). Notably, changes in the Shannon indices in the gut microbiome associated with *Lcn2* loss were more pronounced when compared to our findings in *Gprc5a^−/−^* mice (Figure 2A). We observed a significant increase in alpha diversity as soon as NNK exposure timepoint ended (baseline vs. end of NNK, *p* = 0.0021 of Tukey’s HSD test), followed by a progressive decrease in the Shannon indices to ultimately return to levels lower than that of baseline, by seven months post-NNK (end of NNK vs. seven months post-NNK, *p* = 0.0301 of Tukey’s HSD test; Figure 4A). A similar pattern was noted when probing the alpha diversity of *Gprc5a*^−/−^; *Lcn2*^−/−^ lungs (F-stat = 14.07, *p* = 4.58 × 10^−5^; Figure 4B). Namely, the Shannon indices at the end of NNK exposure were significantly higher when compared to baseline (baseline vs. end of NNK, *p* = 0.0139 of the Tukey HSD test). This notable increase in the Shannon indices in the lungs at the end of tobacco carcinogen exposure was trailed by a swift, significant decrease at three months post-NNK relative to the end of exposure (end of NNK vs. three months post-NNK, *p* = 0.0012), ultimately shifting the alpha diversity of the lungs’ microbiome by the seven months post-NNK timepoint to levels significantly below those observed at baseline (end of NNK vs. seven months post-NNK *p* = 0.001, baseline vs. seven months post-NNK *p* = 0.0131; Figure 4B). Of note, the shift in alpha diversity was more pronounced in the lungs due to the loss of *Lcn2* when contrasted with the gut ecosystem (Figure 4A) and with the *Gprc5a^−/−^* mouse model, which did not demonstrate statistically significant shifts in the lungs’ microbiome alpha diversity (Figure 2B). Hence, it is plausible to surmise that the increased tumor burden in the lungs following the loss of *Lcn2* is closely associated with significant changes in the microbiome of the tumor’s microenvironment. All in all, this suggests that the loss of *Lcn2* is associated with a significant decrease in alpha diversity following exposure to tobacco carcinogen.

The remarkable change in species richness and diversity within the microbial ecosystems of both the lung and the gut following NNK exposure was twinned with marked clustering of the microbiome in each ecosystem (Figure 4C). When comparing the gut microbiome of the two knock-out models (*Gprc5a^−/−^* and *Gprc5a*^−/−^; *Lcn2*^−/−^), apparent clustering is observed along the first axis, indicating clear clustering of the two groups amongst themselves (*p* < 1 × 10^−5^ of the AMOVA test; Figure 4D). Axis two clearly demonstrates a shift in microbial communities due to tobacco carcinogen exposure in the same manner observed in *Gprc5a^−/−^* mice (Figure 4C,D).

These findings highlight the distinct microbial patterns that emerge and progress in association with *Lcn2* expression during lung oncogenesis.

### 2.4. Loss of Lcn2 Provokes Microbial Imbalances Plausibly Related to Increased Tumor Burden

**Figure 5:**
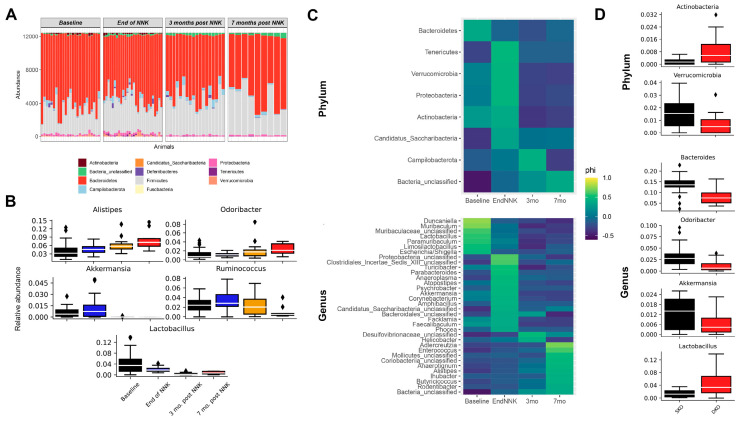
Global fecal microbiome changes associated with *Lcn2* expression during lung oncogenesis. (**A**) Changes in abundance of bacterial phyla at different timepoints of NNK exposure *Gprc5a*^−/−^; *Lcn2*^−/−^. (**B**) Changes in gut select bacterial genera at different timepoints of NNK exposure identified through SIMPER analysis and analyzed via a Kruskal–Wallis test with FDR correction. The corrected *p*-value threshold was 0.05. ♦, Outlier. (**C**) Heat map of the indicator analysis conducted to identify phyla (**upper panel**) and genera (**lower panel**) that are good “indicators” of microbial ecosystems at different NNK exposure timepoints. Indicator species were significantly more abundant and present in all samples belonging to one group and absent or with low abundance in the other group. The *p*-values obtained from the permutations of the indicator analysis were subsequently corrected via FDR procedures. (**D**) Metastats were used to determine phyla and genera that are significantly different in OTU counts between *Gprc5a*^−/−^ and *Gprc5a*^−/−^; *Lcn2*^−/−^ mice. Metastats results were corrected for multiple testing with a false discovery rate (FDR) correction of *p*-values. SKO, single knockout (*Gprc5a*^−/−^); DKO, single knockout (*Gprc5a*^−/−^; *Lcn2*^−/−^). NNK: nicotine-specific nitrosamine ketone. ♦, Outlier.

The increased lung tumors in *Gprc5a*^−/−^; *Lcn2*^−/−^ mice, which are concomitant with widespread changes in the gut and lung microbiomes that are distinct from similarly exposed *Gprc5a^−/−^* mouse littermates, prompted us to further interrogate changes at the phylum and genus levels. Indeed, similar to our findings in *Gprc5a^−/−^* animals, we found significantly and progressively changing bacterial phyla following NNK exposure, including the abolishment of Verrucomicrobia and Actinobacteria in the gut and lung microbial ecosystems (Figure 5A, Supplementary Figures S3A and S4A,B).

Next, we performed a SIMPER analysis and found 24 genera level OTUs that explain dissimilarities among NNK exposure timepoints in the gut of *Gprc5a*^−/−^; *Lcn2*^−/−^ mice, 23 of which remained significant following further analyses (Table 3).

Notably, we observed a significant increase in the abundance of many bacterial genera in the gut microbial ecosystem, including, among others, *Alistipes* and *Odoribacter*, as well as an attenuated abundance of *Akkermansia*, which have been suggested to promote a response to immunotherapy [4], as well as *Lactobacillus* and *Ruminococcus*, whose decreased levels were shown to be associated with various diseases and cancers [18,19,20,21,22,23,24] (Figure 5B, Supplementary Figure S3B). Similar analyses performed on the *Gprc5a*^−/−^; *Lcn2*^−/−^ lung microbial ecosystem identified 27 unique genera through the initial SIMPER analysis, 16 of which remained significant after FDR correction (Table 4).

We then performed an indicator analysis to identify indicator species at various timepoints of tobacco carcinogen exposure in the gut and lungs of *Gprc5a*^−/−^; *Lcn2*^−/−^ mice (Figure 5C, Supplementary Figure S4C). Of particular note, *Escherichia/Shigella* were found to be indicator species at baseline, *Akkermansia* at the end of NNK, *Helicobacter* at three months post-NNK, and *Alistipes* at seven months following exposure to NNK in the gut of *Gprc5a*^−/−^; *Lcn2*^−/−^ mice (Figure 5C).

Given our findings on the distinct clustering of *Gprc5a*^−/−^; *Lcn2*^−/−^ gut microbiome compared to its *Gprc5a*^−/−^ counterpart (Figure 4D) and given the unique genera we unraveled by SIMPER and indicator analyses in *Gprc5a*^−/−^; *Lcn2*^−/−^ mice, we sought to compare differential microbial composition among the two animal genotypes in an attempt to understand the increased tumor burden associated with the loss of *Lcn2*. Indeed, we found baseline differences between the two groups present, irrespective of NNK exposure. For instance, we noted an increased relative abundance of Actinobacteria and decreased Verrucomicrobiota at the phylum level in the *Gprc5a*^−/−^; *Lcn2*^−/−^ mouse model compared to mice with intact *Lcn2* (Figure 4E). Further analyses at the genus levels revealed a decreased baseline abundance of *Bacteroides*, *Odoribacter*, and *Akkermansia* and increased levels of *Lactobacillus* associated with *Lcn2* loss (Figure 4E). The differences observed in the gut microbial composition among the two genotypes persist after exposure to tobacco carcinogen, with changes noted at the phylum and genus levels at the end of NNK and seven months after (Supplementary Figure S5).

Our findings underscore intriguing changes in the host microbiome following exposure to NNK and due to the loss of LCN2, which could explain the previously described increased tumor burden in *Gprc5a*^−/−^; *Lcn2*^−/−^ mice.

## 3. Discussion

An expansive frontier in cancer research fueled by advances in next-generation sequencing is unfolding the imperative roles of microbial niches in oncogenesis and response to therapy [1]. The “polymorphic microbiome” is now identified as a distinctive enabling feature that enables the acquisition of the newly redefined hallmarks of cancer [25]. The roles and abundance of various microbial species that live in symbiosis with the human body heavily depend on fluctuations influenced by several exogenous triggers, such as smoking and diet [8]. Despite advances in therapy, the prognosis of tobacco-associated LUAD remains dismal, warranting new strategies to understand the interplay of the various promoters and initiators driving the pathogenesis of this malignancy [9]. Here, we investigate the evolution of the gut and lung microbiomes following exposure to a tobacco carcinogen. We performed 16S-seq of fecal samples and lung tissues collected prior to and at several time points following NNK exposure and during LUAD development in vivo [10]. We found significant progressive changes in gut and lung microbiome taxonomic composition that are closely associated with the phenotypic development of LUAD. As we previously showed that *Gprc5a*^−/−^ mice with a knock-out of lipocalin 2 (*Gprc5a*^−/−^; *Lcn2*^−/−^*)* are more susceptible to nicotine-specific carcinogen-mediated lung cancer development compared to similarly exposed *Gprc5a*^−/−^ with intact *Lcn2*, we sought to investigate the microbial evolution following NNK exposure in this mouse model [11]. Indeed, we found that loss of *Lcn2*, an antimicrobial protein that is released from host cells during microbiome imbalance and inflammation, was associated with widespread changes in the gut and lung microbiome at all time points tested, distinct from those observed in our *Gprc5a*^−/−^ mouse model, including a decrease in abundance and diversity.

We found significant shifts in alpha and beta diversity following exposure to tobacco carcinogen in both the gut and lung microbial niches of *Gprc5a*^−/−^ and *Gprc5a*^−/−^; *Lcn2*^−/−^ mice that are closely associated with the phenotypic development of LUAD. Following exposure to NNK, we noted a significant decrease in the relative abundance of the phylum Verrucomicrobia, which was previously shown to be associated with increased adverse effects of chemotherapy in lung cancer patients [26]. We also noted a progressive increase in the phylum Firmicutes coupled with a decrease in Bacteroidetes in the gut following exposure to NNK. An increased Firmicutes/Bacteroidetes ratio was demonstrated to be a relevant biomarker of gut dysbiosis and was also shown to mediate gastric and colorectal cancer pathogenesis, as well as response to therapy [27,28,29,30,31,32].

Furthermore, and particularly in *Gprc5a*^−/−^; *Lcn2*^−/−^ mice, we observed a significant decrease in the abundance of the phylum Actinobacteria in both gut and lung microbiomes. This phenomenon resonates with a previous study in which a drastic reduction in Actinobacteria was reported in lung cancer patients compared to healthy controls [33]. This phylum contains many commensal species, which are part of the healthy human microbiome [34]. It is plausible that Actinobacteria are involved in the pathogenesis of lung cancer by means of secondary metabolites, which was demonstrated in a study where isolated *Actinobacteria* sp. from healthy children had compelling anti-tumorigenic potential via secondary metabolites [35].

At the genus level, many human-relevant microbiome changes were noted. Of particular interest, *Akkermansia*, belonging to the phylum Verrucomicrobia, and *Ruminococcus* were significantly decreased following NNK exposure. Both *Akkermansia* and *Ruminococcus* were among the most significantly enriched genera in stool from patients with major pathologic response to immune checkpoint inhibitors relative to non-responders [4,18,19]. Furthermore, levels of *Lactobacillus* were significantly decreased in response to tobacco carcinogen. The anti-tumor effects of *Lactobacillus* sp. were heavily reported in the literature [20,21,22,23,24]. One study found that in patients with superficial bladder cancer, the oral administration of *Lactobacillus casei* positively affected recurrence-free survival rates [36].

Lipocalin 2 (LCN2, human ortholog, neutrophil gelatinase-associated lipocalin [NGAL], also known as siderocalin, 24p3) is an intricate anti-microbial protein of the innate immune system, markedly increased in many inflammatory diseases [37,38,39]. One well-known function of LCN2 is to chelate bacterial siderophores, hence, limiting iron acquisition and subsequent bacterial proliferation [40]. Accordingly, the loss of *Lcn2* may disrupt iron homeostasis in the gut, allowing the growth of facultative pathogenic bacteria over health-promoting intestinal commensals [41]. The ablation of *Lcn2* and exposure to tobacco carcinogen led to increased abundance in the level of *Alistipes* species, which were previously shown to perpetuate disease and promote tumorigenesis [42]. Furthermore, *Gprc5a*^−/−^; *Lcn2*^−/−^ mice had distinct baseline microbiome communities compared to *Gprc5a*^−/−^, irrespective of tobacco carcinogen exposure. For instance, we found that the loss of *Lcn2* was associated with decreased levels of *Akkermansia*, *Odoribacter*, and *Bacteroides*. These findings could partly explain previously described increased tumor burden in *Gprc5a*^−/−^; *Lcn2*^−/−^ mice, as these microbes were previously associated with tumorigenesis and resistance to therapy. For instance, poor responses to the inhibition of CTLA-4 by ipilimumab, as well as increased side effects (such as mucosal damage and colitis), were associated with a decrease in *Bacteroides* species in the intestines [43]. The oral administration of various *Bacteroides* alone or in combination with *Burkholderia cepacia* restored the tumor-suppressing effects of CTLA-4 blockade by augmenting Th1 responses in tumor-draining lymph nodes and promoting intratumoral dendritic cells maturation. Furthermore, the administration of *Bacteroides fragilis* combined with *Burkholderia cepacia* prevented mucosal damage and refractory colitis [44,45,46]. Our overall findings apprise novel pathways implicating antimicrobial host defense mechanisms in the development of tobacco-associated LUAD. We highlight LCN2 as a key player in counteracting LUAD development by possibly maintaining microbiome “eubiosis” (homeostasis and diversity).

We also found that microbial changes were accompanied by significantly decreased gut and circulating levels of SCFAs (propionic and butyric acids) post-tobacco carcinogen exposure and during LUAD development. SCFAs such as butyrate and propionate, secreted by microbes, maintain immune homeostasis by preventing microbial binding to the epithelium [47,48,49,50]. Furthermore, SCFAs, by several mechanisms, maintain an anaerobic environment in the gut that is suitable for the colonization of health-promoting obligate anaerobes [51]. A perturbation in the levels of SCFAs dictates the composition of resident microbial species and subsequent predisposition to carcinogenesis.

Our study is unique for charting changes in the gut and lung microbiomes that are closely associated with the phenotypic evolution of LUAD in a human-relevant mouse model. We found significant progressive changes in microbial species previously known to influence oncogenesis and response to therapy in human lung cancer patients. This work can pave the way for future efforts to unravel the mechanisms behind these microbial changes and their translational relevance. Nonetheless, our study incurred several limitations. NNK was used to induce lung tumor formation in our mice models. However, we acknowledge that other carcinogens released by burned tobacco can affect microbial homeostasis. Hence, further studies are warranted to validate our findings using cigarette smoke and other tobacco-related carcinogens. While most previous efforts were tailored to modulate the gut microbial ecosystem to impact therapeutic responses, focus has now been redirected to target the microbiome residing within the tumor itself to halt cancer progression and impact cancer therapy. Indeed, several studies have highlighted the detrimental effects of the intratumoral microbiome on response to therapy in colorectal and pancreatic cancers [52,53,54]. While our study provides insights into changes in the lung microbial ecosystem in response to tobacco carcinogen, there is still a pressing need to catalog different microbial communities within lung tumors.

Taken together, our findings point towards a novel protective role for microbiome homeostasis in the development of tobacco-associated LUAD. Further studies are warranted to discern which microbiome profiles are likely causally related to tobacco-associated LUAD development and to elucidate the relevance of these key microbial changes among human smokers.

## 4. Materials and Methods

### 4.1. Animal Housing and Tobacco Carcinogen-Exposure Experiments

All animal experiments were conducted in accordance with Institutional Animal Care and Use Committee-approved protocols. The mice were housed and maintained in a pathogen-free animal facility that was approved by the American Association for Accreditation of Laboratory Animal Care. *Gprc5a***^−/−^** and *Gprc5a^−/−^*/*Lcn2^−/−^* mice (C57BL/6) were generated as previously described [10,11]. For tobacco-mediated carcinogenesis experiments, 8-week-old *Gprc5a***^−/−^** and *Gprc5a^−/−^*/*Lcn2^−/−^* mice were divided into four groups (8–10 mice per genotype per time point, matched age, 1:1 male to female ratio) and were injected intraperitoneally with 50 mg/kg of body weight NNK three times per week for eight weeks (150 mg/kg total per week) dissolved in PBS. The mice were sacrificed at baseline (prior to NNK treatment), immediately after the completion of NNK treatment (end of NNK), and at 3 and 7 months following NNK treatment (*Gprc5a***^−/−^** and *Gprc5a^−/−^*/*Lcn2^−/−^*). Lungs and fecal pellets were collected for DNA extraction, 16S rRNA gene amplification, and subsequent DNA sequencing. Blood was also sampled at baseline and seven months following NNK treatment.

### 4.2. Short-Chain Fatty Acid Extraction

To determine the relative abundance of short-chain fatty acids in mouse plasma, whole blood, and stool samples, extracts were prepared and analyzed by ultra-high-resolution ion-chromatography-mass spectrometry (IC-MS) with a Dionex ICS-5000+ system interfaced with an Orbitrap Fusion Tribrid mass spectrometer. Metabolites were extracted using ice-cold 0.1% ammonium hydroxide in methanol: water (80:20). Samples were centrifuged at 17,000× *g* for 5 min at 4 °C, and supernatants were transferred to clean tubes, followed by evaporation to dryness under nitrogen. The samples were reconstituted in deionized water, then 5 μL was injected into a Thermo Scientific Dionex ICS-5000+ IC system containing a Thermo IonPac AS11 250×2 mm 4 μm column. The flow rate was 300 µL/min at 35 °C, and the gradient conditions were as follows: 0–24 min, 1–15 mM of KOH; 24–40 min, 15–60 mM of KOH; 40–48 min, 60–100 mM of KOH; 48–58 min, 99 mM of KOH; 58–60 min, 1 mM of KOH. To improve desolvation and sensitivity, methanol was added post-column by an external pump via a low dead volume mixing tee. Data were acquired using a Thermo Orbitrap Fusion Tribrid Mass Spectrometer under ESI negative mode at a resolution of 240,000 (FWHM at m/z 200) for MS1 acquisition. Raw files were imported into Thermo Trace Finder software for final analysis.

### 4.3. Total DNA Isolation from Mouse Lung Tissues and Fecal Pellets

Total DNA was isolated from homogenized tissues (mouse lungs and fecal pellets) using the Qiagen RNeasy mini kit (Qiagen) and DNeasy PowerSoil Pro Kit soil, respectively, according to the manufacturer’s recommendations. Extracted DNA was quantitated via QuBit fluorometric assay (ThermoFisher Scientific; Waltham, MA, USA) and normalized to 15ng/ul as appropriate.

### 4.4. DNA Sequencing and Analysis

Library preparation of the V4 variable region of the 16S rRNA gene utilizing Kozich et al., 2013 dual-indexed approach was carried out on all samples, including a positive mock community standard, an extraction control, and a negative amplification control. After library normalization and pooling, the final library pool was subjected to quality control measures utilizing the 2100 BioAnalyzer (Agilent; Santa Clara, CA, USA). The concentration of the library pool was determined by a qPCR using the KAPA Library Quantification Kit for Next Generation Sequencing (KAPA Biosystems; Woburn, MA, USA). Sequencing-by-synthesis was performed on the MiSeq System utilizing v2 chemistry in a paired-end 250-bp fashion, resulting in 32.4 million reads passing filter. The de-multiplexing of raw sequence data and FASTQ generation was carried out using bcl2fastq (v2.17) via Illumina BaseSpace Sequencing Hub. Sequence reads pertaining to the 16S V4 region were subjected to quality control and filtering according to processes previously described elsewhere [55,56]. Briefly, forward and reverse reads were overlapped, producing sequence contigs. After the overlap of the two reads, there were 11,544,748 resulting sequence contigs. Sequences that contained ambiguous bases and contigs longer than 275 base pairs were removed. The reads were aligned to a trimmed version of the SILVA v132 database, which included the 16S gene region, to confirm that all reads overlapped the same sequenced V4 variable region. The reads were pre-clustered, grouping reads that varied by only two nucleotides. The VSEARCH algorithm was utilized to identify potential chimeric reads, which were subsequently removed from the data set. The sequences were then classified using a Bayesian classifier trained on the Ribosomal Database Project (RDP) database (version 18). After classification, the sequences that were classified as non-bacterial were removed. After this extensive quality control process, the sequences were used to create a distance matrix representing the uncorrected pairwise distances between all unique sequences. This distance matrix was the input to the Opti-Clust OTU clustering method used to cluster OTUs at a 97% (species) similarity cutoff. The OTU clustering process resulted in 3350 species-level OTUs. OTUs were classified based on the consensus taxonomy of all sequences in a specific OTU. An analysis of the mock community sample included in the sequencing run determined that the error rate after quality control was 0.000033%. In addition to the de novo OTU clustering approach, the sequences were also binned into phylotypes based on the sequence classification to test for differentially abundant features at various taxonomic levels (phylum and genus levels).

### 4.5. Statistical Analysis

In order to explore alpha diversity, Shannon indices were calculated using the species-level OTUs. Differences in alpha diversity among the various time points were investigated using an ANOVA and Tukey’s post hoc test where necessary. The Bray–Curtis dissimilarity matrix, generated using species-level OTUs, was used as an input to the principal coordinate analysis to visualize the data. Clustering observed in the data was tested for statistical significance using an AMOVA with 10,000 iterations. A SIMPER analysis was used to determine the taxa contributing to the dissimilarity observed between the samples [57,58]. For ease of interpretation, the SIMPER analysis was carried out at the genus and phylum levels to identify genera and phyla that explain at least 1% of the dissimilarity observed between our groups of interest. Although the SIMPER analysis identifies taxa that explain the variation in dissimilarity observed within the dataset, it does not determine statistical significance. For this reason, the taxa identified through the SIMPER analysis were also analyzed via a Kruskal–Wallis test with an FDR correction. The corrected *p*-value threshold was 0.05. For the genotype group comparisons, Metastats was used to determine whether there is a significant difference in OTU counts between any of the exposure time points after SIMPER. The Metastats results were also corrected for multiple testing with a false discovery rate (FDR) correction of *p*-values. An indicator analysis with 10,000 permutations was conducted to identify genera that are good “indicators” of microbial ecosystems associated with particular NNK exposure time points (R package). The *p*-values obtained from the permutations were subsequently corrected via FDR procedures.

## Figures and Tables

**Table 1:** *Gprc5a^−/−^* gut microbiome SIMPER analysis.

Timepoint Comparison	Number of OTUS Explaining Greater Than 1%	Cumulative Dissimilarity Explained (%)
Baseline vs. end of NNK	21	89.8
End of NNK vs. three months post-NNK	20	89.5
Three months post-NNK vs. seven months post-NNK	21	90.5
Baseline vs. three months post-NNK	20	91.1
Baseline vs. seven months post-NNK	22	92.7
End of NNK vs. seven months post-NNK	22	90.5

**Table 2:** *Gprc5a^−/−^* lung microbiome SIMPER analysis.

Timepoint Comparison	Number of OTUS Explaining Greater Than 1%	Cumulative Dissimilarity Explained (%)
Baseline vs. end of NNK	23	70
End of NNK vs. three months post-NNK	22	68
Three months post-NNK vs. seven months post-NNK	22	79.4
Baseline vs. three months post-NNK	22	70
Baseline vs. seven months post-NNK	22	76.9
End of NNK vs. seven months post-NNK	22	78.1

**Table 3:** *Gprc5a*^−/−^; *Lcn2*^−/−^ gut microbiome SIMPER analysis.

Timepoint Comparison	Number of OTUS Explaining Greater Than 1%	Cumulative Variation Explained
Baseline vs. end of NNK	21	88.4
End of NNK vs. three months post-NNK	23	88.7
Three months post-NNK vs. seven months post-NNK	21	89.7
Baseline vs. three months post-NNK	18	87.8
Baseline vs. seven months post-NNK	16	86
End of NNK vs. seven months post-NNK	23	88.6

**Table 4:** *Gprc5a*^−/−^; *Lcn2*^−/−^ lung microbiome SIMPER analysis.

Timepoint Comparison	Number of OTUS Explaining Greater Than 1%	Cumulative Variation Explained
Baseline vs. end of NNK	24	65.2
End of NNK vs. three months post-NNK	20	69.5
Three months post-NNK vs. seven months post-NNK	14	81.9
Baseline vs. three months post-NNK	18	70.2
Baseline vs. seven months post-NNK	16	78.2
End of NNK vs. seven months post-NNK	17	74.4

## Data Availability

Data will be made available to the GEO by the time of publishing.

## Supplementary Materials

The supporting information can be downloaded at: https://www.mdpi.com/article/10.3390/ijms231810930/s1.
