# Supplementary material for: Pathogenesis of Tobacco-Associated Lung Adenocarcinoma Is Closely Coupled with Changes in the Gut and Lung Microbiomes
Source: Int J Mol Sci. 2022 Sep 18;23(18):10930. doi: 10.3390/ijms231810930 (PMC9502774; doi:10.3390/ijms231810930)
Supplement: Supplementary file 1 [file ijms-23-10930-s001.zip › ijms-1889248-supplementary.pdf]

A

Phylum level

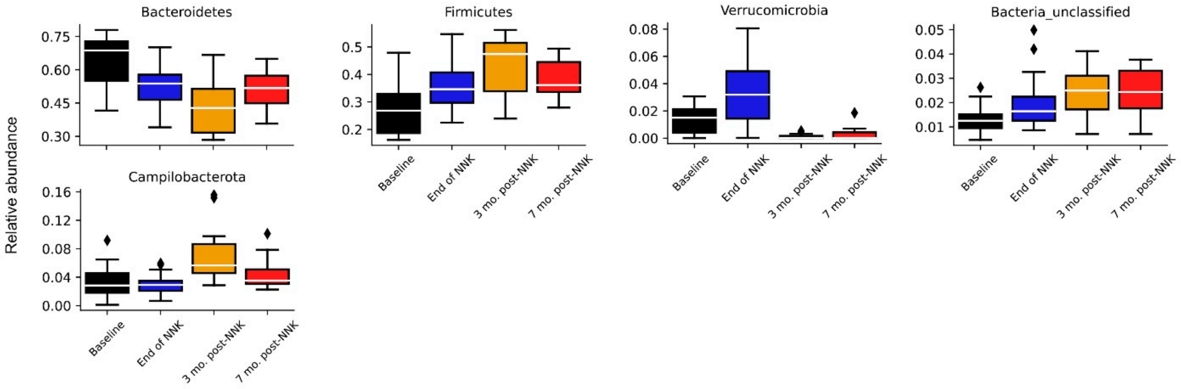

B

Genus level

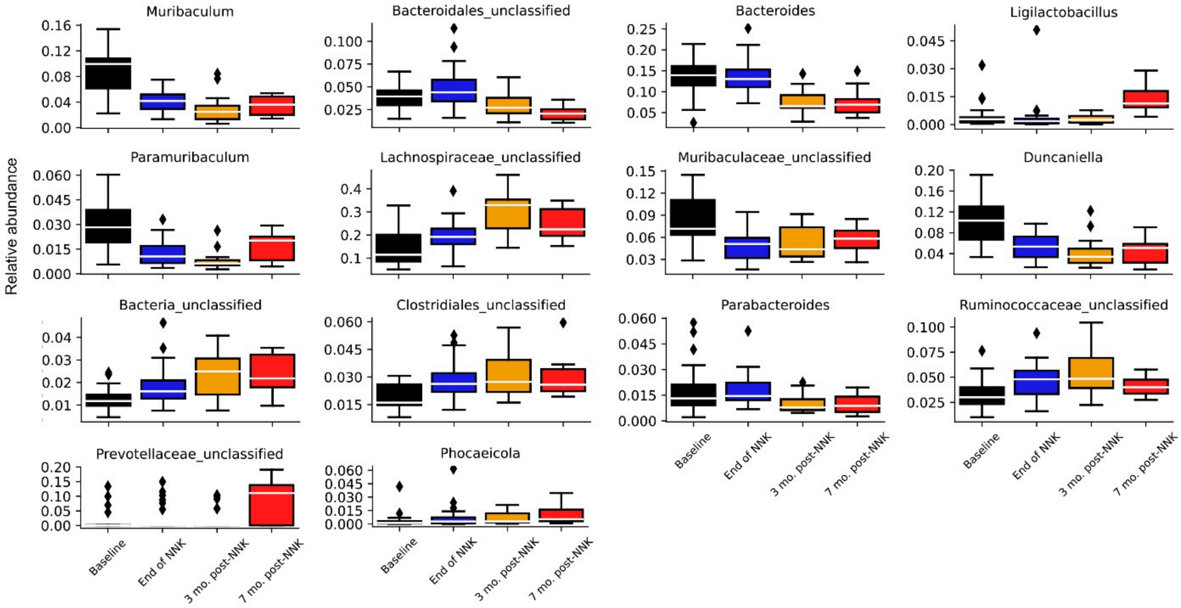

Supplementary Figure S1. Significantly changing phyla and genera during the phenotypic evolution of tobacco carcinogen-associated LUAD in *Gprc5a*$^{-/-}$ animals. A. Changes in gut bacterial phyla at different timepoints of NNK exposure identified through SIMPER analysis and analyzed via a Kruskal-Wallis with FDR correction. The corrected *P*-value threshold was 0.05. **B.** Changes in gut bacterial genera at different timepoints of NNK exposure identified through SIMPER analysis and analyzed via a Kruskal-Wallis with FDR correction. The corrected *P*-value threshold was 0.05. NNK: nicotine-specific nitrosamine ketone. ♦, Outlier.

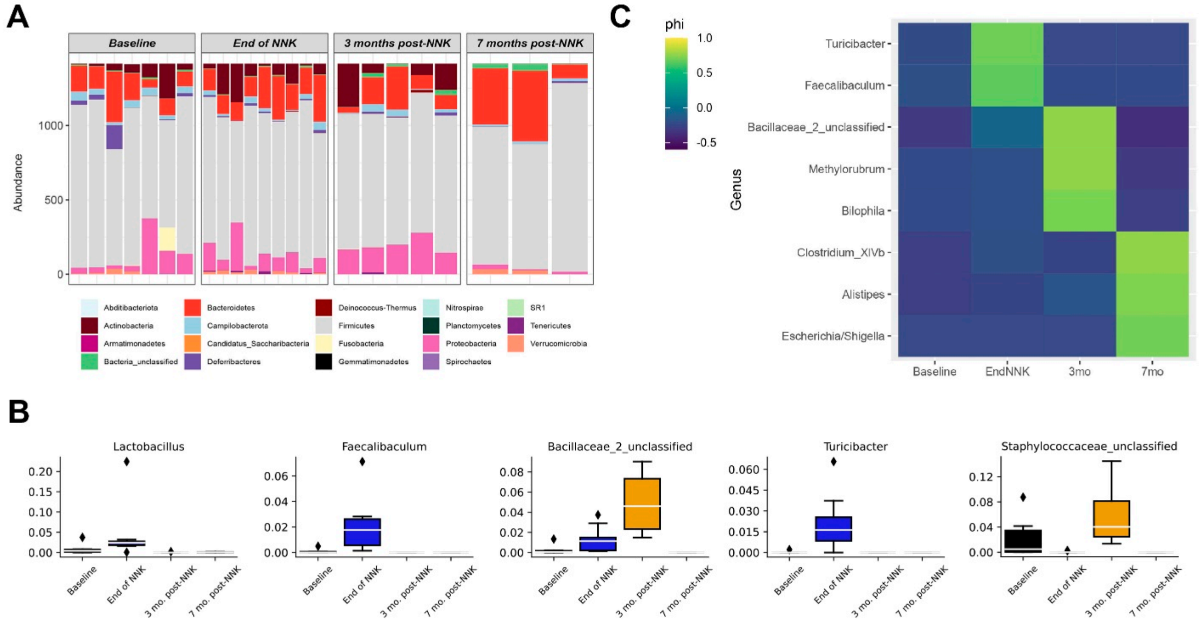

Supplementary Figure S2. Shifts in the lung microbial ecosystem during LUAD pathogenesis in *Gprc5a*$^{-/-}$ animals. A. Changes in abundance of lungs' bacterial phyla at different timepoints of NNK exposure. **B.** Changes in lungs' bacterial genera at different timepoints of NNK exposure identified through SIMPER analysis and analyzed via a Kruskal-Wallis with FDR correction. The corrected *P*-value threshold was 0.05. ♦, Outlier. **C.** Heat map of the indicator analysis conducted to identify genera (right) that are good "indicators" of the lung microbiome at different NNK exposure timepoints. Indicator species were significantly more abundant and present in all samples belonging to one group and absent or with low abundance in the other group. The *P*-values obtained from the permutations of the indicator analysis were subsequently corrected via FDR procedures.

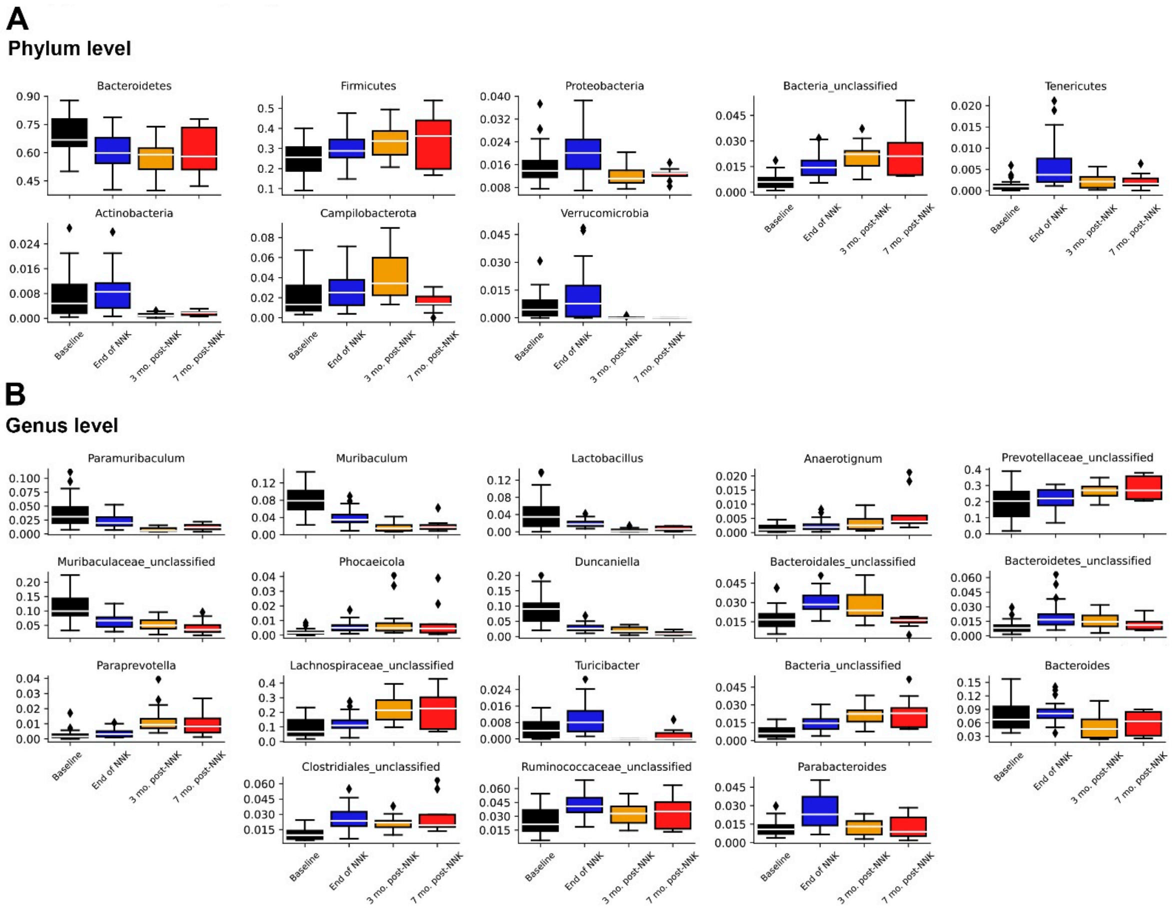

Supplementary Figure S3. Significantly changing phyla and genera during the phenotypic evolution of tobacco carcinogen-associated LUAD in *Gprc5a*$^{-/-}$ *;Lcn2*$^{-/-}$ animals. A. Changes in gut bacterial phyla at different timepoints of NNK exposure identified through SIMPER analysis and analyzed via a Kruskal-Wallis with FDR correction. The corrected *P*-value threshold was 0.05. **B.** Changes in gut bacterial genera at different timepoints of NNK exposure identified through SIMPER analysis and analyzed via a Kruskal-Wallis with FDR correction. The corrected *P*-value threshold was 0.05. NNK: nicotine-specific nitrosamine ketone. ♦, Outlier.

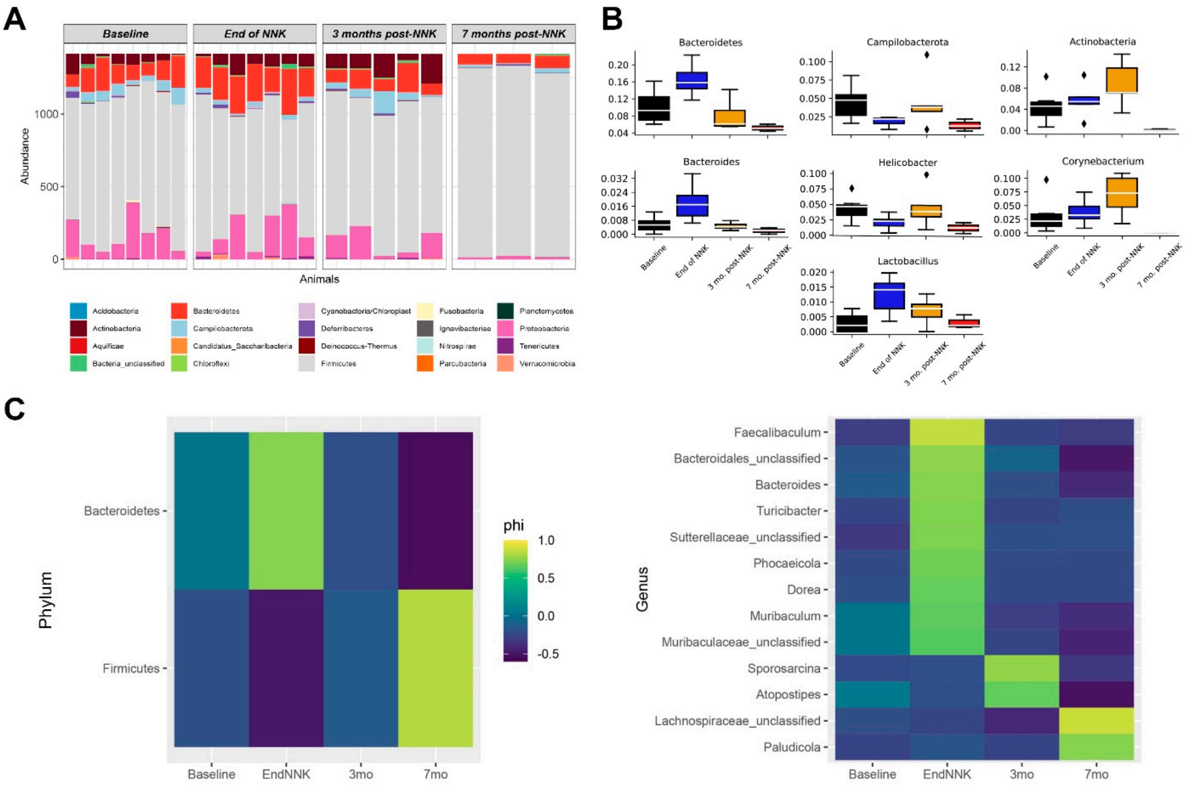

Supplementary Figure S4. Shifts in the lung microbial ecosystem during LUAD pathogenesis in *Gprc5a*$^{-/-}$ *;Lcn2*$^{-/-}$ animals. A. Changes in abundance of lungs' bacterial phyla at different timepoints of NNK exposure. **B.** Changes in lungs' select bacterial genera at different timepoints of NNK exposure identified through SIMPER analysis and analyzed via a Kruskal-Wallis with FDR correction. The corrected *P*-value threshold was 0.05. ♦, Outlier. **C.** Heat map of the indicator analysis conducted to identify phyla (left) and genera (right) that are good "indicators" of the lung microbiome at different NNK exposure timepoints. Indicator species were significantly more abundant and present in all samples belonging to one group and absent or with low abundance in the other group. The *P*-values obtained from the permutations of the indicator analysis were subsequently corrected via FDR procedures.

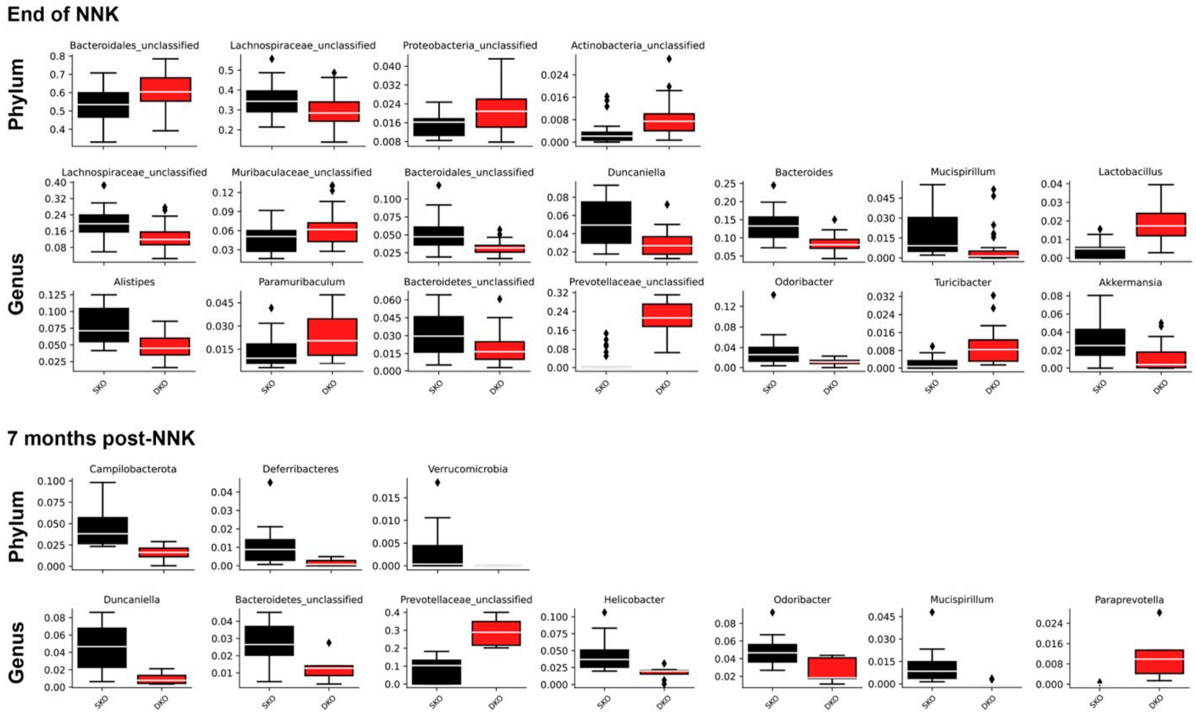

Supplementary Figure S5. Loss of *Lcn2* is associated in with global gut microbiome changes before and after tobacco exposure. Metastats were used to determine phyla and genera that are significantly different in OTU counts between *Gprc5a*$^{-/-}$ and *Gprc5a*$^{-/-}$;*Lcn2*$^{-/-}$ mice at end of NNK and seven-month post-NNK. Metastats results were corrected for multiple testing with a false discovery rate (FDR) correction of *P*-values. SKO, single knock-out (*Gprc5a*$^{-/-}$); DKO, single knock-out (*Gprc5a*$^{-/-}$;*Lcn2*$^{-/-}$). NNK: nicotine-specific nitrosamine ketone. ♦ Outlier.
